# Case Report: Integrated physiological and performance profiling of an elite athlete with differences of sex development

**DOI:** 10.3389/fspor.2026.1906817

**Published:** 2026-10-19

**Authors:** Ke Hu, Kaiyun Huang, Xiao Xu, Blair R. Hamilton, Sandra Rozenštoka, Fabio Pigozzi, Samuel Ka-Kin Ling, Ada S. Cheung, Eric Vilain, Yannis P. Pitsiladis

**Affiliations:** 1 Centre for Exercise Science and Medicine (CESAME), Hong Kong Baptist University, Hong Kong, Hong Kong SAR, China; 2 Department of Sport and Health Sciences, Hong Kong Baptist University, Hong Kong, Hong Kong SAR, China; 3 Department of Biology, Faculty of Science, Hong Kong Baptist University, Hong Kong, Hong Kong SAR, China; 4 BGI Research Shenzhen, Shenzhen, China; 5 School of Sport and Exercise Science, Institute of Sport, Manchester Metropolitan University, Manchester, United Kingdom; 6 Riga Stradins University, Riga, Latvia; 7 Sports Laboratory, International Federation of Sports Medicine (FIMS) Collaborating Centres of Sports Medicine (CCSM), Riga, Latvia; 8 Latvian Sports Medicine Association, Riga, Latvia; 9 International Federation of Sports Medicine, Fédération Internationale de Médecine du Sport (FIMS), Lausanne, Switzerland; 10 European Federation of Sports Medicine Associations, Lausanne, Switzerland; 11 Department of Movement, Human and Health Sciences, University of Rome “Foro Italico”, Rome, Italy; 12 Villa Stuart Sport Clinic, FIFA Medical Center of Excellence, Rome, Italy; 13 Department of Orthopaedics and Traumatology, Faculty of Medicine, The Chinese University of Hong Kong, Hong Kong, Hong Kong SAR, China; 14 Department of Medicine, The University of Melbourne, Parkville, VIC, Australia; 15 Institute for Clinical and Translational Science, University of California, Irvine, CA, United States

**Keywords:** differences of sex development (DSD), dihydrotestosterone, endocrinology, sex classification, sports genomics, SRD5A2, testosterone

## Abstract

**Background:**

Recent developments in athlete eligibility frameworks, including sex-determining region Y (SRY) gene screening, have renewed focus on the biological basis of sex classification in sport. However, the relationship between genetic and endocrine markers and sport-specific performance remains incompletely understood, particularly in athletes with differences of sex development (DSD).

**Objective:**

To demonstrate a multidimensional, sport-specific framework for evaluating elite athletes with DSD, in whom genetic, endocrine and performance characteristics may not align uniformly with binary sex categories.

**Methods:**

An integrated multidimensional physiological assessment was conducted in an elite football athlete with DSD. Assessments included endocrine profiling, whole-genome sequencing, body composition, neuromuscular performance, pulmonary function, aerobic capacity, and sport-specific field testing. Results were benchmarked against published elite football datasets and in-house comparator cohorts using z-scores.

**Results:**

The 46,XY DSD athlete with a homozygous pathogenic SRD5A2 variant (c.578A > G) and a markedly elevated T:DHT ratio demonstrated heterogeneous physiological and football-specific performance characteristics across domains. Sprint ability and explosive power were above elite female football references, while strength and aerobic capacity were broadly comparable with female reference values.

**Conclusion:**

This case illustrates the value of evaluating athletes using sport-specific physiological and performance data rather than isolated biological markers alone. Accumulation of similar multidimensional datasets may help inform more evidence-based, sport-specific approaches to athlete evaluation.

## Highlights

### What is already known on this topic

Athletic performance and eligibility in athletes with differences of sex development (DSD) remain highly debated, yet physiological evidence in elite athletes with DSD is extremely limited. Existing policies and scientific discussions are largely informed by indirect evidence, population comparisons or endocrine markers rather than integrated sport-specific physiological profiling.

### What this study adds

This study provides a rare multidimensional physiological assessment of an elite athlete with DSD, integrating genomic, endocrine, body composition, neuromuscular, cardiorespiratory and sport-specific performance measures within a single athlete. The findings demonstrate that biological, endocrine and performance characteristics may not align uniformly and support the importance of multidimensional physiological assessment.

### How this study might affect research, practice or policy

These findings support the need for larger sport-specific physiological datasets to inform evidence-based eligibility frameworks in women's sport. They also highlight the importance of case-by-case physiological evaluation rather than reliance on isolated biological markers when interpreting performance-related biology in elite athletes.

## 1 Introduction

Differences of sex development (DSD) comprise a heterogeneous group of conditions in which chromosomal, gonadal, endocrine, and phenotypic sex characteristics do not align in a uniform way (1). This heterogeneity makes it difficult to infer sport-specific physiological phenotype from isolated biological markers alone. Although the sex-determining region Y (SRY) gene is a key initiator of testicular differentiation (2), downstream phenotypic expression depends on broader genomic, gonadal, endocrine, and receptor-level pathways. The direct evidence linking biological classification markers to sport-specific physiological phenotype remains limited (3, 4). This gap is especially relevant in football, where performance depends on multiple interacting domains, including body composition, explosive power, strength, aerobic capacity, and repeated-sprint ability. Recent eligibility frameworks in female sport have renewed attention to this issue, particularly through the use of biological markers such as SRY (5). Accordingly, the present investigation demonstrates a multidimensional, sport-specific framework for evaluating an elite football athlete with DSD, integrating molecular, endocrine, physiological and football-specific performance data rather than relying on isolated biological markers alone.

### 1.1 Case presentation

The participant was a 20-year-old Ghanaian elite football athlete competing at national-team level. Before enrolment in the present study, she had not undergone formal genetic assessment. She reported no chronic disease, current symptoms, allergies, medication or other therapy, or supplement use. Her medical history included a right medial knee-ligament strain in 2025 that did not restrict testing; she had no acute illness or injury at the time of assessment and was able to complete maximal exercise testing. Detailed family history and structured psychosocial history were not collected as part of the performance-focused study protocol and were therefore unavailable for this report. As part of the present investigation, whole-genome sequencing demonstrated sex-chromosome read-depth patterns consistent with a 46,XY chromosomal complement and confirmed the presence of the SRY locus. Genomic analysis identified an SRD5A2 finding associated with 5*α*-reductase type 2 deficiency. No therapeutic intervention was administered. All planned assessments were completed without adverse or unanticipated events. Because the investigation was cross-sectional, no post-assessment clinical follow-up or longitudinal outcomes were collected. To protect participant confidentiality, the athlete's club, playing position and other potentially identifying sporting details are not reported. Throughout the manuscript, the participant is referred to as an elite football athlete competing in women's football; 46,XY refers specifically to her chromosomal complement, and genomic and endocrine characteristics are reported separately and explicitly.

## 2 Methods

To contextualise the physiological and performance profile of this elite football athlete, comparisons were undertaken against both published football datasets and in-house reference cohorts. Published datasets from 25 national-level female football players (6) and 29 national-level male football players were used as the primary sport-specific performance references (7). These datasets were selected on the basis of high protocol comparability, including similar dynamometry procedures, contraction velocities, range of motion settings, and performance testing methodologies, thereby allowing meaningful benchmarking of football-specific neuromuscular characteristics (6). The in-house comparator groups of recreationally active adults, including cisgender men (CM, *n* = 9), cisgender women (CW, *n* = 8), and transgender women (TW, *n* = 9), were used as contextual and methodological reference groups. The TW group was included as a contextual comparator, acknowledging that these populations differ in developmental history, diagnosis, and physiology. Training load was not adjusted for, as no validated method exists to normalise performance outcomes relative to training exposure in this context.

The athlete underwent comprehensive clinical, genetic, endocrine, physiological and sport-specific performance assessments. A summary of the sequence of evaluations included in this report is presented in Table 1.

**Table 1:** Timeline of clinical, genomic and physiological assessment.

Timepoint	Event
Elite football career	Participation in elite-level football competition
Clinical assessment	Evaluation for differences of sex development (DSD)
Genetic testing	WGS findings consistent with a 46,XY chromosomal complement
Endocrine assessment	Hormonal and biochemical profiling
Physiological assessment	DXA body composition, pulmonary function, strength and aerobic capacity testing
Performance assessment	Repeated sprint ability (RSA), sprint testing and Yo-Yo IR2 testing
Current study	Integrated physiological and performance profiling completed and reported

A comprehensive hormonal profile was determined from fasting venous blood sampling. Total testosterone and oestradiol were quantified by liquid chromatography–tandem mass spectrometry (LC-MS/MS), while sex hormone-binding globulin, luteinizing hormone, follicle-stimulating hormone, progesterone, androstenedione, and free testosterone were quantified by the clinical laboratory panel. Dihydrotestosterone (DHT) was additionally quantified by the external reference laboratory that processed the sample. Total testosterone, DHT, free testosterone, and androstenedione were reported in nmol·L⁻^1^; values originally supplied in mass units were converted to molar units using the analyte-specific molecular mass. The T:DHT ratio was calculated after expressing total testosterone and DHT in the same molar unit. DNA was extracted from EDTA-treated blood (BD Vacutainer® K2EDTA Blood Collection Tubes, BD Life Sciences, USA) for whole genome sequencing.

Whole-body composition and skeletal integrity were subsequently evaluated using dual-energy x-ray absorptiometry (DXA; Hologic Horizon A, USA) at standard scan mode. The participant was positioned supine on the scanning bed according to the manufacturer's guidance. A single whole-body scan was performed to derive regional and total body composition variables, followed by site-specific scans of the lumbar spine (L1-L4) and left proximal femur, from which areal bone mineral density (g·cm⁻^2^) was determined using manufacturer-defined regions of interest.

Neuromuscular performance testing comprised explosive, isometric, and isokinetic strength assessments performed on the dominant leg. These parameters were specifically chosen for their relevance to the sport of football. Countermovement jump (CMJ) performance was assessed using a force platform (Swift SpeedMat, Swift Performance Equipment, Australia), with the participant completing three maximal efforts separated by 20-second passive recovery. Peak jump height (cm), derived from flight-time methodology was retained as the primary explosive performance outcome, and the highest value was used for analysis.

Maximal voluntary isometric knee extensor torque was subsequently assessed using an isokinetic dynamometer (Humac Norm, Computer Sports Medicine Inc, USA). The participant performed three maximal voluntary isometric contractions at a fixed knee joint angle of 120° (180° represents full extension), each lasting 5 s, with 30-second rest intervals provided between trials to minimize fatigue, and the highest peak torque value retained for analysis (8). Subsequently, isokinetic assessments were performed through a controlled range of motion of 78° (knee angle 90-168°), with testing conducted for the knee extensors followed by the knee flexors to ensure consistency in muscle group sequencing. Concentric and eccentric contractions were assessed at angular velocities of 60°·s⁻^1^ followed by 180°·s⁻^1^, with four maximal repetitions performed at each velocity and a 20-second rest interval between sets to limit fatigue accumulation (6). Peak torque values were recorded for all conditions and normalized to body mass (Nm·kg⁻^1^) for subsequent analysis.

Pulmonary function testing was conducted to characterize the mechanical aspects of ventilation using integrated spirometry and body plethysmography systems (Vyntus ONE and Vyntus BODY, CareFusion, Germany) operated via SentrySuite software (version 3.10.6). Spirometric measurements were first obtained using the Vyntus ONE system, including forced vital capacity (FVC) and forced expiratory volume in one second (FEV₁). Upon completion of spirometry, the participant was transitioned to the Vyntus BODY system for assessment of static lung volumes using whole-body plethysmography.

Aerobic capacity was examined using a ramp protocol (9) performed on a motorized treadmill (h/p cosmos pulsar 3p, Germany). The participant wore a mask and head straps (Hans Rudolph, USA), and inspired and expired gases were measured by O₂ and CO₂ analysers in the flowmeter sample line using breath-by-breath analysis (Sensormedics Vmax Encore 229d, USA). The participant was monitored at rest for approximately 5 min until respiratory exchange values had stabilized (respiratory quotient ≈ 0.80; resting V˙O₂ ≈ 500 mL·min⁻^1^). Heart rate was measured continuously (Frontier X2, Fourth Frontier, USA) at the end of each stage to verify maximal effort.

Two high-intensity intermittent exercise tests were conducted on an outdoor 5-a-side football pitch. Ambient heat stress was quantified using wet-bulb globe temperature (WBGT; 5400 Heat Stress Tracker, Kestrel, USA). Repeated sprint ability (RSA) was assessed using a standardized protocol consisting of 6 maximal sprints over a distance of 30 m separated by an active recovery interval of 20 s. Sprint times were recorded using electronic timing gates (Brower TCi Two Gate System, Brower, USA) positioned at start and finish line. Performance indices included best sprint time, mean sprint time, and percentage performance decrement. Yo-Yo Intermittent Recovery Test Level 2 (Yo-Yo IR2) commenced at an initial running speed of 13.0 km·h⁻^1^ and progressing through incremental speed stages dictated by pre-recorded audio cues. The test was terminated when the participant failed to reach the finish line on two consecutive occasions, with total distance covered recorded as the primary outcome measure.

Whole-genome sequencing was performed on the MGI DNBSEQ-T1 + platform (2 × 150 bp paired-end reads) to an average depth of 51.3  ×  . Raw sequencing data underwent standard quality-control processing, alignment to the human reference genome (GRCh38), and germline variant calling using an established bioinformatics pipeline incorporating BWA-MEM, Picard, and GATK HaplotypeCaller. Homozygous and heterozygous mutations were identified using a custom R script, based on inference from Variant Allele Frequency (VAF). Genome-wide copy number profiling was performed using read-depth methods (R QDNAseq package) with a 1 Mb bin size. Sex-chromosome read-depth patterns and alignment to the sex-determining region Y (SRY) locus were additionally examined to assess chromosomal sex context. All 5,551,776 single-nucleotide variants and small insertions/deletions were annotated using the Ensembl Variant Effect Predictor (VEP, v115), with integration of ClinVar clinical significance annotations (CLNSIG, CLNREVSTAT, CLNDN; ClinVar version 2026-08-29), SIFT and PolyPhen in silico predictions, and population allele frequencies. A genome-wide ClinVar pathogenic or likely pathogenic (P/LP) filter was first applied. Two published targeted DSD gene panels were subsequently evaluated: the M014 panel (171 genes) and the KY043 panel (267 genes) (10). Variants of interest were visually reviewed using Integrative Genomics Viewer (IGV, v2.19.7).

All statistical analyses were conducted using Jamovi (Version 2.6.26.0; The Jamovi Project, Sydney, Australia). Given the single-athlete design, analyses were framed as normative comparisons. For each physiological and performance variable, the DSD athlete's observed value was positioned relative to the empirical distribution of the relevant comparator groups using standardized z-scores. Training load was not included as a covariate because no prespecified and defensible functional form relating training exposure to the present outcomes was assumed; adjustment under these conditions was considered more likely to introduce model-dependent bias than improve inference. Accordingly, interpretation focused on the magnitude, direction, and practical physiological relevance of deviations across domains rather than dichotomous statistical significance.

### 2.1 Equity, diversity and inclusion statement

This study was conducted in accordance with principles of equity, diversity and inclusion (EDI). Given the rarity and sensitivity of differences of sex development (DSD) in elite sport, the study was designed to ensure respectful, confidential and athlete-centred evaluation. Clinical, physiological and genomic assessments were interpreted within an individualised framework and without assumptions based solely on sex-related biological characteristics. The study aimed to contribute evidence to an underrepresented area of sports medicine research while recognising the diversity and complexity of athlete biology. More broadly, understanding how genetic, endocrine and physiological variation contributes to elite athletic performance remains a fundamental challenge in human performance and enhancement science.

## 3 Results

### 3.1 Participant characteristics, endocrine profile, and molecular findings

Participant and comparator-group descriptive characteristics are presented in Table 2. Endocrine results are presented in Table 3. The athlete was aged 20 years, with a height of 1.63 m, body mass of 60.1 kg, BMI of 22.6 kg·m⁻^2^, and reported training load of 9,813 MET·min·wk⁻^1^. Endocrine profiling demonstrated total testosterone of 23.6 nmol·L⁻^1^ and DHT of 0.80 nmol·L⁻^1^; when expressed in the same molar units, these values yielded a testosterone-to-dihydrotestosterone ratio of 29.5:1 (23.6/0.80). LH was 22.0 U·L⁻^1^ and FSH was 34.7 U·L⁻^1^. Oestradiol was 243.7 pmol·L⁻^1^, progesterone was 1.20 nmol·L⁻^1^, and androstenedione was 5.1 nmol·L⁻^1^. To further characterise the biological context of this endocrine profile, whole-genome sequencing was performed (Supplementary Figures S1–S4). Sequencing findings were consistent with a 46,XY chromosomal complement and confirmed the presence of the SRY locus. A homozygous missense variant was identified in the SRD5A2 gene (NM_000348.4: c.578A > G; p.Asn193Ser; chr2:31529427 T > C, GRCh38), classified as pathogenic in ClinVar (VCV000459638), and predicted deleterious by SIFT (score 0) and probably damaging by PolyPhen-2 (score 0.987). This variant is associated with 5*α*-reductase type 2 deficiency, characterized by impaired conversion of testosterone to dihydrotestosterone. Whole-genome ClinVar screening identified six variants classified as pathogenic or likely pathogenic (Supplementary Table S1): SRD5A2 (chr2:31529427, c.578A > G, missense), HPS1 (chr10:98427229, frameshift), HBB (chr11:5227002, missense), CDKN2B (chr9:22003368, 3'UTR), SERPINA7 (chrX:106036488, missense), and XG (chrX:2748343, upstream variant). Of these, only the SRD5A2 variant is known to cause DSD or androgen metabolism disorders; the remaining five are unrelated to the athlete's clinical phenotype. Systematic evaluation of the M014 and KY043 DSD gene panels (Supplementary Table S2) identified no additional pathogenic or likely pathogenic variants beyond the SRD5A2 finding.

**Table 2:** Descriptive characteristics of the athlete with DSD and comparator groups.

Variable	DSD	Elite Female (*n* = 25)	Elite Male (*n* = 29)	CM (*n* = 9)	CW (*n* = 8)	TW (*n* = 9)
Age (y)	20	20 (3)	24 (4)	36 (6)	32 (7)	36 (8)
Height (m)	1.63	1.69 (0.05)	1.81 (0.05)	1.79 (0.1)	1.68 (0.1)	1.77 (0.1)
Body mass (kg)	60.1	64.5 (9.4)	79.6 (7)	79.4 (8.2)	66.9 (18.0)	78.0 (11.7)
Training load	9,813	(-)	(-)	4,027 (2,481)	4,052 (2,224)	4,332 (4,247)

Comparator values are presented as mean (SD). The athlete is presented under the column DSD as a single observed value. Training load in MET·min·wk⁻^1^.

**Table 3:** Endocrine profile of the athlete with DSD.

Variable	DSD	Laboratory Reference Interval	Unit
Oestradiol	243.7	follicular <529; ovulatory 235–1309; luteal 205–786; postmenopausal <118	pmol·L⁻^1^
Progesterone	1.20	Follicular: <4.45; ovulatory: 1.5–5.5; luteal: 10.62–81.28; postmenopausal: <2.32	nmol·L⁻^1^
Luteinizing hormone (LH)	22.0	1.0–52.2	U·L⁻^1^
Follicle-stimulating hormone (FSH)	34.7	Follicular: 2.5–10.2; ovulatory: 3.4–33.4; luteal: 1.5–9.1; postmenopausal: 23–116.3	U·L⁻^1^
Androstenedione	5.1	2.44–15.05	nmol·L⁻^1^
Total testosterone	23.6	0.29–1.67	nmol·L⁻^1^
Free testosterone	0.30	0.010–0.066	nmol·L⁻^1^
Sex hormone-binding globulin (SHBG)	68.0	17.69–138.26	nmol·L⁻^1^
Dihydrotestosterone (DHT)	0.80	<0.7	nmol·L⁻^1^

Endocrine results are presented for the athlete only. Female phase-specific reference intervals were provided by the reporting laboratory. Androstenedione was originally reported as 1.46 ng/mL and converted to 5.1 nmol·L⁻^1^.

### 3.2 Body composition, physiological and performance characteristics

Body composition, bone mineral density and selected physiological outcomes are presented in Table 4 as original observed values. The DSD athlete, who was younger than comparator groups, demonstrated higher bone mineral density, low adiposity, and intermediate lean mass relative to the in-house comparator cohorts. Since the DSD athlete was scanned using a Hologic Horizon system whereas comparator groups were scanned using a GE Lunar Prodigy system, cross-calibrated values were also calculated (11). The athlete's lumbar spine BMD increased from 1.37 to 1.63 g/cm^2^, total hip BMD increased from 1.16 to 1.30 g/cm^2^, fat mass decreased from 11.60 kg to 8.62 kg, and relative skeletal muscle mass index (RSSMI) increased from 8.80 to approximately 9.83 kg/m^2^ after conversion.

**Table 4:** Outcomes relative to in-house dataset.

Variable	DSD	CM	z	CW	z	TW	z
LS BMD	1.37	1.19 (0.14)	1.29	1.17 (0.10)	2.04	1.17 (0.12)	1.73
TH BMD	1.16	1.16 (0.16)	0.03	1.01 (0.07)	2.16	1.02 (0.11)	1.30
Fat Mass	11.60	18.16 (4.39)	−1.50	19.19 (8.78)	−0.86	22.63 (4.92)	−2.24
FFM	48.70	58.13 (9.38)	−1.01	43.17 (7.56)	0.73	52.52 (7.60)	−0.50
BF%	19.20	21.08 (5.46)	−0.34	24.80 (6.91)	−0.81	28.06 (3.49)	−2.54
VAT	29.60	58.11 (36.42)	−0.78	24.88 (23.63)	0.20	58.33 (39.20)	−0.73
RSSMI	8.80	9.05 (1.18)	−0.21	7.50 (0.64)	2.04	8.04 (0.81)	0.94
Isometric	203	283 (68)	−1.18	193 (63)	0.17	250 (54)	−0.86
CMJ (cm)	41.9	32.7 (5.9)	1.56	21.6 (3.6)	5.60	24.1 (4.7)	3.76
FVC	2.72	5.06 (0.66)	−3.55	4.18 (0.78)	−1.86	4.84 (0.65)	−3.25
FEV_1_	2.55	4.09 (0.59)	−2.63	3.47 (0.60)	−1.53	3.79 (0.53)	−2.33
FEV_1_/FVC ratio	0.94	0.81 (0.04)	3.11	0.83 (0.04)	2.78	0.78 (0.04)	3.75
Absolute V˙O₂max	2.93	4.05 (0.57)	−1.96	3.01 (0.43)	−0.19	3.41 (0.52)	−0.92
Relative V˙O₂max	48.7	52.67 (6.52)	−0.61	46.38 (6.35)	0.37	44.33 (3.24)	1.35

DSD values represent the participant's observed values. Comparator group data are presented as mean (SD), with corresponding z-scores calculated relative to each comparator group. CM = cisgender men; CW = cisgender women; TW = transgender women; LS BMD = lumbar spine bone mineral density; TH BMD = total hip bone mineral density; FFM = fat-free mass; BF% = body fat percentage; VAT = visceral adipose tissue; RSSMI = relative skeletal muscle mass index.

When compared with elite football cohorts (Figure 1A), the athlete's bone mineral density, body fat percentage, and fat-free mass were within the reported range for elite female football players (12). Relative to elite male football squads, the athlete's body composition was characterized by higher body fat percentage and lower absolute lean mass (13).

**Figure 1:**
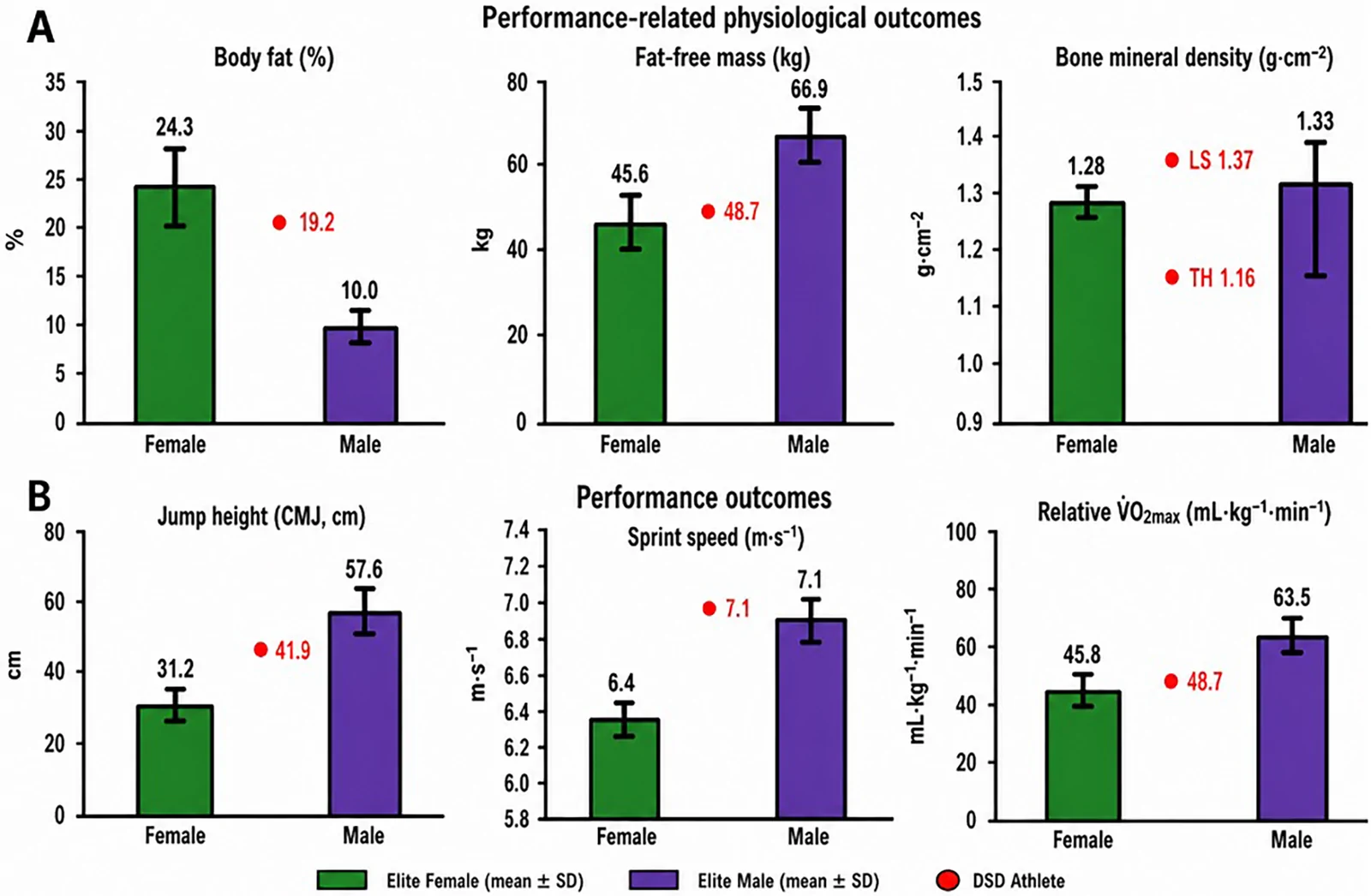
Key indicators relative to elite football reference groups. Bars show comparator-group mean ± SD; red markers show the DSD athlete's observed values. Female reference data were from national-level Finnish soccer players (6). Male DXA reference data were derived from English Premier League (13). Male CMJ and VO₂max reference data were derived from the Norwegian elite division (7), whereas male sprint data were derived from a first-division German Bundesliga club (19). Comparator cohorts were not directly matched and are presented for contextual interpretation.

### 3.3 Strength and power

Countermovement jump height was 41.9 cm in the DSD athlete. Compared with the female football comparator cohorts, this value was higher than both the U18 group mean of 30.0 ± 2.8 cm and the First Team group mean of 32.3 ± 3.4 cm, corresponding to large positive z-scores (4.25 and 2.82, respectively). Compared with the male football position-specific reference groups, the athlete's CMJ was lower than the defence mean of 55.1 ± 6.5 cm (z = −2.03), the midfield mean of 50.5 ± 4.4 cm (z = −1.95), and the attack mean of 57.6 ± 5.1 cm (z = −3.08). Thus, CMJ performance was above female comparator means and below male reference values (Figure 1B).

CMJ height was higher than all in-house comparator-group means, with z-scores of 5.60 relative to CW, 3.76 relative to TW and 1.56 relative to CM (Table 4). In contrast, isometric peak knee-extensor torque was 203 Nm and showed a different pattern: the athlete's value was close to the CW mean (z = 0.17) but lower than the CM and TW means (z = −1.18 and −0.86, respectively). These findings indicate dissociation between explosive performance and maximal isometric force production.

Knee extensor torque showed variability across contraction modes and velocities relative to the elite female football reference cohorts (Table 5, Figure 2). Under concentric conditions, extensor torque at 60°·s⁻^1^ was similar to both the U18 and first-team means (*z* = 0.67 and 0.00, respectively). At 180°·s⁻^1^, concentric extensor torque was higher than the U18 reference (*z* = 2.00) and equivalent to the first-team mean (*z* = 0.00). Eccentric extensor torque was lower than both comparator cohorts at both velocities. Knee flexor torque showed a similarly mixed profile.

**Table 5:** Isokinetic outcomes relative to elite female football comparator cohorts.

Variable	DSD	U18	z_U18	First Team	z_First Team
Econ60	2.9	2.5 (0.6)	0.67	2.9 (0.3)	0.00
Eecc60	2.6	4.0 (0.5)	−2.80	4.5 (0.8)	−2.38
Econ180	2.1	1.5 (0.3)	2.00	2.1 (0.3)	0.00
Eecc180	1.7	3.6 (0.4)	−4.75	4.2 (0.9)	−2.78
Fcon60	2.3	1.4 (0.3)	3.00	1.2 (0.2)	5.50
Fecc60	2.2	1.8 (0.4)	1.00	1.7 (0.3)	1.67
Fcon180	1.6	1.1 (0.3)	1.67	1.0 (0.2)	3.00
Fecc180	1.3	1.9 (0.3)	−2.00	1.7 (0.3)	−1.33

DSD values represent the participant's observed values. Comparator group data are presented as mean ± SD, with corresponding z-scores calculated relative to each comparator group. These z-scores are descriptive normative comparisons; no hypothesis tests or statistical-significance thresholds were applied.

**Figure 2:**
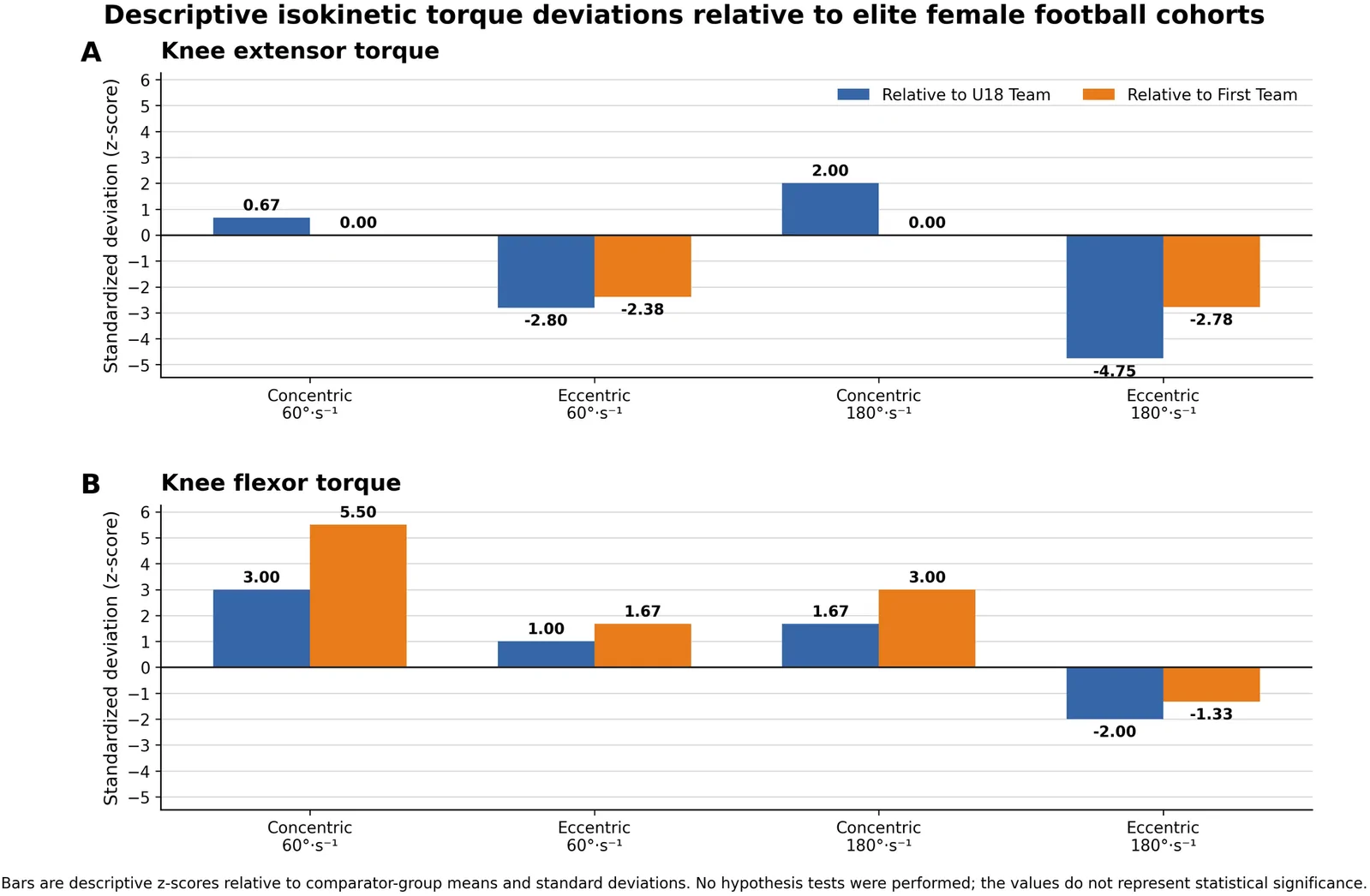
Descriptive standardized deviations in concentric and eccentric knee extensor and flexor torque at 60 and 180°·s⁻^1^ relative to the U18 team and first team comparator cohorts (6). Bars represent z-scores calculated from comparator-group means and standard deviations. No hypothesis tests were performed, and the figure contains no statistical-significance markers.

### 3.4 Lung function and aerobic capacity

Forced vital capacity (FVC; 2.72 L) and forced expiratory volume in one second (FEV₁; 2.55 L) were lower than all comparator group means (Table 4). When expressed relative to predicted values, however, FVC and FEV₁ corresponded to 85% and 90% of predicted, respectively. The largest negative deviations for FVC were observed relative to CM (z = −3.55) and TW (z = −3.25), with smaller deviations relative to CW. A similar pattern was observed for FEV₁, with the athlete lower than all groups and the largest deviations relative to CM (z = −2.63) and TW (z = −2.33). In contrast, the FEV₁/FVC ratio was higher than all comparator means, with the largest positive deviations observed relative to TW (z = 3.75) and CM (z = 3.11), indicating preserved expiratory flow relative to lung volume.

The athlete's relative V˙O₂max was 48.7 mL·kg⁻^1^·min⁻^1^ and was slightly higher than the selected elite female football reference means. Her maximal running speed was 15.7 km·h⁻^1^,corresponding to z = 1.09 relative to U18 players and z = 0.45 relative to First Team players. Compared with male football position-specific reference groups, relative V˙O₂max was lower than the reported means for defenders (61.5 ± 3.3 mL·kg⁻^1^·min⁻^1^, z = −3.88), midfielders (66.4 ± 5.7 mL·kg⁻^1^·min⁻^1^, z = −3.11), and attackers (63.5 ± 3.5 mL·kg⁻^1^·min⁻^1^, z = −4.23). Male football reference data for maximal running speed were not available (Figure 1B).

Ambient environmental conditions were monitored throughout testing, with wet-bulb globe temperature (WBGT) recorded at 27.79 °C, indicating moderate environmental heat stress. Heart rate increased progressively during the protocol, peaking at 169 beats·min⁻^1^ at test termination. The athlete completed 440 m on the Yo-Yo Intermittent Recovery Test Level 2, corresponding to level 19:4 at 17.0 km·h⁻^1^, then reached level 20:1 at 17.5 km·h⁻^1^, although receiving two warnings and subsequently did not complete the stage. The estimated V˙O₂max was 51.3 mL·kg⁻^1^·min⁻^1^. Relative to elite football comparator cohorts (Figure 1B), the best 30 m sprint time recorded for the athlete was faster than both first-team players (4.63 ± 0.18 s; z = −2.28) and U18 players (4.74 ± 0.12 s; z = −4.33).

## 4 Discussion

### 4.1 Principal findings

The present study provides an integrated multidimensional physiological and performance profile of an elite football athlete with DSD. The findings demonstrated substantial heterogeneity across physiological domains rather than a uniformly male-typical profile. Sprint and explosive performance exceeded elite female football reference means and approached some male football reference values, whereas aerobic capacity, pulmonary function, and several strength measures aligned more closely with female reference cohorts or remained intermediate. At the molecular level, whole-genome sequencing confirmed a homozygous pathogenic SRD5A2 variant (c.578A > G; p.Asn193Ser) as the underlying cause of 5*α*-reductase type 2 deficiency. Genome-wide ClinVar screening identified six pathogenic or likely pathogenic variants (Supplementary Table S1), but only the SRD5A2 variant is known to cause DSD or androgen metabolism disorders. Systematic evaluation of the M014 and KY043 DSD gene panels (Supplementary Table S2) confirmed no additional pathogenic or likely pathogenic variants beyond the SRD5A2 mutation. Collectively, these findings illustrate that genotype, endocrine profile, and sport-specific physiological phenotype may not align uniformly within an individual athlete.

### 4.2 Physiological interpretation

Endocrine assessment showed total testosterone of 23.6 nmol·L⁻^1^ and DHT of 0.80 nmol·L⁻^1^, yielding a T:DHT molar ratio of 29.5:1. This ratio reflects disproportionately low DHT relative to testosterone and is compatible with impaired 5*α*-reductase activity (14). The homozygous pathogenic SRD5A2 variant (c.578A > G; p.Asn193Ser) identified in this athlete provides a molecular basis for this biochemical profile, as 5*α*-reductase type 2 — encoded by SRD5A2 — is the principal enzyme responsible for the peripheral conversion of testosterone to dihydrotestosterone. Previous reports likewise indicate that SRD5A2-related DSD can coexist with high-level sporting performance while remaining phenotypically variable (14–17). Together with elevated LH and FSH, this profile did not align with typical female or eugonadal male endocrine profiles (18). However, the present data do not allow inference that the endocrine profile directly explains the relative differences observed between sprint, jump, isometric, eccentric, or aerobic outcomes. Accordingly, the most defensible interpretation is descriptive: this athlete exhibited a heterogeneous performance profile across domains, the mechanistic basis of which cannot be determined in the present study.

Training exposure represents an important contextual factor when interpreting these findings. The athlete's self-reported activity volume was approximately 2.3-2.4 times the means of the in-house recreationally active comparator groups, and training load was not statistically adjusted due to the absence of a validated method for normalisation in this single-athlete context. This difference likely contributes to the observed advantages in strength and power relative to the in-house cohorts. However, most core performance outcomes—including CMJ, isokinetic strength, VO₂max and body composition—were compared against published elite football cohorts with comparable training loads. Comparisons against these training-matched cohorts also demonstrated substantial heterogeneity across performance domains, with sprint and explosive performance approaching male reference values while aerobic capacity and several strength measures aligned with female cohorts. This cross-domain heterogeneity is not readily explained by training load alone, as a uniformly well-trained athlete would be expected to show more consistent advantages across all domains relative to recreationally active controls. A smaller set of secondary outcomes (pulmonary function, visceral adipose tissue, skeletal muscle index) were only available against in-house cohorts due to limited published data in elite football populations, and these should be interpreted with additional caution.

The neuromuscular profile of the athlete was characterised by relative strengths across selected performance domains. Isokinetic assessment likewise showed variability across contraction modes and velocities, which should be interpreted cautiously given the potential influence of familiarity, task specificity, and football-related training exposure on dynamometer performance. CMJ height was higher than elite female football reference means (6), while the magnitude of this difference is uncertain due to the absence of position-matched data. CMJ height was lower than the available elite male football reference means (7). This difference was particularly evident when the comparison was restricted to position-specific male attackers. Results from field tests may provide the most sport-relevant context for interpreting this case. The athlete's 30 m sprint time was faster than the elite female reference groups (6) and closely aligned with the professional male first-division reference values (19). Low fatigue index and sprint decrement support strong repeated-effort tolerance and efficient between-sprint recovery (20–22).

Beyond its relevance to DSD, the present study contributes to broader questions regarding the biological determinants of elite athletic performance. Human enhancement research has traditionally focused on exogenous interventions, training methodologies and technological innovations. However, understanding how naturally occurring biological variation influences performance phenotype is equally important for advancing scientific understanding of human performance capacity.

### 4.3 Clinical and policy implications

Pulmonary and aerobic findings further support this broader interpretation. Although absolute spirometry volumes were lower than those of all comparison groups, FVC and FEV₁ corresponded to 85% and 90% of predicted values, respectively, indicating relatively small absolute lung volumes. The elevated FEV₁/FVC ratio further suggested preserved expiratory flow relative to lung volume. Relative V˙O₂max showed an intermediate pattern: the athlete's laboratory-measured value was slightly above elite female football reference means (6) but lower than the available male position-specific football reference value (7). Her Yo-Yo IR2 performance fell within the reported range for elite female cohorts and below available male reference values (23, 24). The ∼5% difference between laboratory-measured V˙O₂max and Yo-Yo IR2-estimated V˙O₂max likely reflects the distinction between direct gas-exchange measurement and field-based performance estimation (25).

Within the in-house comparisons, the athlete's DXA profile was characterised by elevated lumbar spine bone mineral density, low adiposity, and intermediate-to-high RSSMI. Cross-calibration results strengthened, rather than changed, the interpretation: GE-equivalent BMD and RSSMI were higher than the uncorrected Hologic values, while GE-equivalent fat mass was lower. Thus, the athlete's profile of high skeletal indices, comparatively high RSSMI and low adiposity was retained after correction. But the original observed values were retained as the primary reported data to avoid over-reliance on correction equations not directly validated in the present sample. The in-house strength and pulmonary data also showed heterogeneity. Isometric knee extensor torque was closely aligned with cisgender women but lower than cisgender men and transgender women, indicating that the athlete's elevated jump performance was not accompanied by proportionally elevated maximal static knee extensor torque. However, the in-house groups were not matched to the athlete for age, training status, competition level, or sport. Therefore, they are most useful for contextualising laboratory-derived physiology rather than defining sport-specific performance level. The present findings highlight the importance of multidimensional physiological assessment when interpreting eligibility-related biology in elite sport. Football performance is highly multifactorial and cannot be reliably inferred from isolated chromosomal, genetic, or endocrine markers alone. While this single case cannot determine eligibility policy, it demonstrates that genotype, endocrine status, and performance phenotype may not align uniformly within individual athletes. These findings support the need for larger sport-specific physiological datasets to inform evidence-based eligibility frameworks in women's sport.

### 4.4 Limitations

Several methodological and ethical limitations should be considered when interpreting the present findings.

First, this single-athlete, cross-sectional case report cannot support population-level inference or establish a performance phenotype attributable to 46,XY DSD, the SRD5A2 finding or the endocrine profile. The standardized z-scores describe the athlete's distance from comparator-group means and are not inferential tests. They are sensitive to the means, standard deviations and sample sizes of the source cohorts. The design also prevents causal separation of androgen biology, developmental history, maturation, training, coaching and other individual characteristics.

Second, the clinical context was incomplete. Detailed family history and structured psychosocial history were not collected as part of the performance-focused protocol. Consequently, possible familial patterns, the athlete's broader psychosocial context and the personal effects of genetic testing and diagnosis could not be assessed. Genetic testing was first performed during this study; without family history or family genetic data, the inheritance and segregation of the SRD5A2 finding could not be evaluated. These omissions limit the clinical and psychosocial interpretation of the case.

Third, comparator-dataset heterogeneity is a major limitation. The published reference data were collected from Finnish, Norwegian, English and German cohorts between 1998 and 2023 and represented different competition levels, including national-level female players, elite male players, position-specific groups, English Premier League players and German first-division players. The in-house groups were recreationally active adults rather than elite football athletes. The cohorts differed from the Ghanaian national-team athlete in nationality, sporting environment, age, training history, selection criteria and competition level. Differences in era may also reflect changes in training practice, professionalisation, testing technology and athlete preparation. Equipment, procedures and testing conditions were not identical across all outcomes. The z-scores therefore locate the athlete within each source distribution and are not directly interchangeable across datasets.

Fourth, training exposure and measurement differences add uncertainty. The athlete's training exposure was self-reported in MET·min·wk⁻^1^ rather than football-specific GPS load, session-RPE, heart-rate-based training impulse or verified training records, and comparable training-exposure data were unavailable for the published elite football cohorts. Training load could not be adjusted adequately in a single-athlete analysis, and applying effect estimates from differently trained populations and protocols would introduce false precision. The study employed a two-pronged comparator design: published elite football cohorts provided training-comparable sport-specific references but with limited outcome ranges, while in-house cohorts provided comprehensive phenotyping but differed in training exposure. DXA measurements were obtained on a different scanner platform from the in-house comparator data, and familiarity, equipment and protocol differences may also have affected the dynamometry, jump, pulmonary and field-test comparisons.

Fifth, the specificity of the reported information creates a residual risk of deductive re-identification. Although direct identifiers were removed, the combination of the athlete's age, Ghanaian national-team context, rare SRD5A2-related DSD, endocrine and genomic findings, and detailed performance profile may permit identification by people familiar with the athlete. This risk arises from the combined information rather than from any single endocrine measurement. Reporting therefore followed a data-minimisation approach: club, playing position and other non-essential contextual details were withheld, and genomic findings were reported only to the level required for scientific interpretation. The residual risk limits both the detail that can be reported and the extent to which participant-level data can be shared.

## 5 Conclusion

In this athlete, endocrine and genomic findings provided a clear DSD context, while physiological and football-specific testing showed a heterogeneous performance profile across domains. This observation may be particularly relevant in sports such as football, where performance depends on multiple interacting domains rather than a single physiological trait. These findings illustrate the value of multidimensional, sport-specific physiological assessment when evaluating athlete phenotype in elite sport. Multidimensional physiological profiling in elite athletes with DSD remains particularly underrepresented in the literature.

## 6 Athlete perspective

The athlete participated voluntarily in this study and consented to publication of the findings. The athlete considered the comprehensive physiological assessment valuable in improving understanding of their individual biological and performance characteristics. The athlete hopes that studies of this nature will contribute to a more evidence-based understanding of differences of sex development in sport.

## Data Availability

No public repository deposition is planned for the raw whole-genome sequencing data or the complete individual-level dataset because the combination of demographic, sporting, genomic, endocrine, physiological and performance information creates a substantial risk of deductive re-identification in this single-athlete case. Requests for limited additional de-identified data should be directed to the corresponding author, Yannis Pitsiladis (ypitsiladis@hkbu.edu.hk), and will be considered individually..
