# Supplementary material for: Case Report: Integrated physiological and performance profiling of an elite athlete with differences of sex development
Source: Front Sports Act Living. 2026 Oct 19;8:1906817. doi: 10.3389/fspor.2026.1906817 (PMC13724393; doi:10.3389/fspor.2026.1906817)
Supplement: Supplementary file 1 [file Datasheet1.docx]

***Supplementary Figure 1*** . Heterozygous and homozygous variant distribution across autosomes and sex chromosomes.

The fraction of heterozygous (het) and homozygous (hom) variant calls was compared across autosomes and the non-pseudoautosomal regions (non-PAR) of the sex chromosomes. Autosomes show 57.9% heterozygous and 42.1% homozygous variants (n = 5,165,579 called sites), consistent with diploid inheritance. The X chromosome non-PAR region shows 0.5% heterozygous and 99.5% homozygous variants (n = 136,823 called sites), and the Y chromosome non-PAR region shows 2.7% heterozygous and 97.3% homozygous variants (n = 8,833 called sites). The near-absence of heterozygous variants on both sex chromosomes reflects hemizygosity. This pattern confirms the presence of a single X and a single Y chromosome, consistent with a 46,XY karyotype.

***Supplementary Figure 2***. Genome-wide copy number profile across chromosomes.

Genome-wide copy number profiling was performed using read-depth methods (R QDNAseq package, 1 Mb bin size). Autosomes (chromosomes 1-22) cluster around log2 ratio = 0, consistent with diploid copy number (2 copies), whereas the X and Y chromosomes show values near log2 ratio = −1, consistent with single-copy (hemizygous) representation. These findings independently support a 46,XY chromosomal complement.

***Supplementary Figure 3.*** Normalized copy‑number profile of the SRY locus on chromosome Y.

Normalized copy number at the SRY locus (chrY:2,786,855-2,787,842; Yp11.2) demonstrates a single-copy signal (≈1), confirming the presence of the sex-determining region Y (SRY) gene on the Y chromosome. No duplication or deletion of the SRY locus was detected. This finding is consistent with the 46,XY karyotype and confirms the presence of the SRY gene.

***Supplementary Figure 4***. SRD5A2 gene structure and IGV visualization of the c.578A>G (p.Asn193Ser) homozygous mutation.

The SRD5A2 gene (NM_000348.4) comprises five exons, with the start codon (ATG) in exon 1 and the stop codon (TAA) in exon 5. The homozygous c.578A>G (p.Asn193Ser) mutation is located within exon 4 (chr2:31,529,427 T>C, GRCh38). IGV visualization confirms homozygosity, with all 63 sequencing reads supporting the variant allele (C). This variant results in an asparagine-to-serine substitution at amino acid position 193 and is classified as pathogenic in ClinVar (VCV000459638).
